# *Saccharomyces cerevisiae* nutrient signaling pathways show an unexpected early activation pattern during winemaking

**DOI:** 10.1186/s12934-020-01381-6

**Published:** 2020-06-06

**Authors:** Beatriz Vallejo, Emilia Matallana, Agustín Aranda

**Affiliations:** Institute for Integrative Systems Biology, I2SysBio, University of Valencia-CSIC, Parc Cientific UV. Av. Agustín Escardino 9, Paterna, 46980 Valencia, Spain

**Keywords:** *Saccharomyces cerevisiae*, Wine, Nutrient signaling pathways, TORC1, Snf1, Nitrogen Catabolite Repression

## Abstract

**Background:**

*Saccharomyces cerevisiae* wine strains can develop stuck or sluggish fermentations when nutrients are scarce or suboptimal. Nutrient sensing and signaling pathways, such as PKA, TORC1 and Snf1, work coordinately to adapt growth and metabolism to the amount and balance of the different nutrients in the medium. This has been exhaustively studied in laboratory strains of *S. cerevisiae* and laboratory media, but much less under industrial conditions.

**Results:**

Inhibitors of such pathways, like rapamycin or 2-deoxyglucose, failed to discriminate between commercial wine yeast strains with different nutritional requirements, but evidenced genetic variability among industrial isolates, and between laboratory and commercial strains. Most signaling pathways involve events of protein phosphorylation that can be followed as markers of their activity. The main pathway to promote growth in the presence of nitrogen, the TORC1 pathway, measured by the phosphorylation of Rps6 and Par32, proved active at the very start of fermentation, mainly on day 1, and ceased soon afterward, even before cellular growth stopped. Transcription factor Gln3, which activates genes subject to nitrogen catabolite repression, was also active for the first hours, even when ammonium and amino acids were still present in media. Snf1 kinase was activated only when glucose was exhausted under laboratory conditions, but was active from early fermentation stages. The same results were generally obtained when nitrogen was limiting, which indicates a unique pathway activation pattern in winemaking. As PKA remained active throughout fermentation, it could be the central pathway that controls others, provided sugars are present.

**Conclusions:**

Wine fermentation is a distinct environmental situation from growth in laboratory media in molecular terms. The mechanisms involved in glucose and nitrogen repression respond differently under winemaking conditions.

## Background

The yeast *Saccharomyces cerevisiae* plays a successful dual role as both a biotechnological tool and a model organism for basic research. It is the main organism behind the production of some fermented foods like alcoholic beverages and bread. *S. cerevisiae* efficiency relies on its ability to adapt its metabolism to whatever carbon source the growth substrate may offer, and to cope with adverse environmental conditions. In winemaking, the substrate is rich in hexoses, such as glucose and fructose, and fermentative metabolism does not only produce ethanol, but also other metabolites required to obtain balanced wine [1]. *S. cerevisiae* is highly tolerant to the ethanol it produces, which prevents other, less tolerant, microorganisms from growing. The ability to sense environmental conditions, and to trigger an efficient adaptive response without preventing proliferation and without diminishing fermentative metabolism, is one of the key elements for yeast technological success [2].

Nutrient sensing and signaling pathways lie at the core of *S. cerevisiae’s* ability to adapt to changing environments, and these pathways have been exhaustively described, and even first discovered, in the laboratory strains of this microorganism [3–5]. A variety of molecular systems responds to the presence or absence of nutrients, and most are cross-regulated to achieve a coordinate metabolic response. However, there are two key players in growth and proliferation when the main nutrients (i.e., carbon and nitrogen sources) are present, namely glucose-induced cAMP-dependent protein kinase A (PKA) and the nitrogen-sensing Target Of Rapamycin (TOR) pathway. PKA is a cAMP-activated kinase that represses stress tolerance and adaptation mechanisms, and stimulates fermentation and cell proliferation [4]. cAMP is produced by adenylate cyclase, stimulated mainly by Ras G-proteins. TORC1 senses intracellular nitrogen availability, particularly the mobilization of amino acids from the vacuole [6]. All amino acids are able to stimulate TORC1 activity, but leucine has a stronger impact, probably due to a particular mechanism involving leucyl-tRNA synthetase in direct TORC1 regulation [7]. Glutamine is a key molecule in nitrogen metabolism, and it also has specific mechanisms to activate TORC1 [8]. Similarly, many inhibitors that induce amino acid starvation also trigger TORC1 inhibition, and they do so in specific ways. For instance, methionine sulfoximine (MSX) inhibits glutamine synthetase to cause intracellular glutamine starvation that triggers the inactivation of most TORC1 functions. However, it does not have the same signature as the inhibition of the pathway by the drug rapamycin, which targets the core of TORC1 activity [9]. TORC1 has many targets, like protein kinase Sch9, which controls protein synthesis, as well as many downstream branches that control different aspects of amino acids metabolism and transport, including the system called Nitrogen Catabolite Repression (NCR) [5]. Preferred nitrogen sources, like glutamine and ammonium, that repress the use of non preferred sources, e.g. proline or urea. GATA transcription factors Gln3 and Gat1, which stimulate the import and catabolism of other nitrogen sources, are broadly repressed by TORC1 action, but this is a fine-tuned system that reacts differently to distinct stimuli, like nitrogen starvation, rapamycin, MSX, etc. [10]. Additional, but related, pathways respond to specific nutritional deficits. The Gcn2 kinase senses amino acid starvation to promote amino acid biosynthesis [4]. When glucose is exhausted, Snf1 kinase is activated to promote the use of other carbon sources. Therefore, Snf1 lies at the core of events known as glucose repression where the presence of glucose shuts down respiration, as well as the use of non fermentable carbon sources like glycerol, but also of alternative sugars and gluconeogenesis [4]. Snf1 is also able to phosphorylate Gln3 in some circumstances [11]. Thus Snf1 seems to play a role in the coordination of both types of repression.

Grape juice is generally regarded as a rich growth medium that meets all the nutritional requirements of wine yeasts. However, imbalances and deficiencies can happen and lead to stuck or sluggish fermentations, which are a major concern for the wine industry [12]. Global nitrogen limitation is the most usual nutritional deficiency to take place during winemaking, as grape juice is rich in sugars, but relatively poor in ammonium and amino acids [1]. Addition of ammonium salts is the conventional technological intervention to supplement nitrogen-deficient musts. This is why long-lasting interest has been shown in mechanisms that allow yeast cells to use other less favorable nitrogen sources, normally controlled by NCR. The transcription of the genes coding general amino acid transporter *GAP1* and ammonium permease *MEP2* is repressed before ammonium is depleted in the medium, which indicates that NCR operates during grape juice fermentation [13]. Arginase activity also increases when nitrogen is consumed. Overall, the transcriptional changes produced by entry into the stationary phase due to nitrogen exhaustion fit the TORC1 inactivation pattern [14]. However, the transcriptomic analysis of wine yeast mutants in NCR elements confers an unexpected complexity of the NCR response during winemaking [15]. A mutant with constitutively alleviated NCR shows that only 13 of the 80 described genes repressed by NCR increase transcription during grape juice fermentation. After adding diammonium phosphate, 34 of the known NCR genes were down-regulated, so the mechanisms described under laboratory conditions cannot be taken for granted under industrial conditions. Other nutrients have often been overlooked, but may influence metabolism as much as nitrogen can, particularly when unbalanced. For instance, yeast viability reduces in the presence of excess nitrogen during lipid-limited fermentations [16]. This effect is alleviated when the gene coding for TORC1-controlled kinase Sch9 is deleted, which indicates that this nitrogen-triggered cell death is controlled by the activity of nutrient-sensing pathways. Similarly, carbon-to-nitrogen ratios may be sensed by yeast through these pathways to promote a coordinated response. Diminishing the effects of TORC1 by deleting *SCH9* extends chronological longevity in a laboratory medium that is rich in nitrogen and poor in carbon, while it shortens the life span when sugars are abundant and nitrogen is scarce, as under winemaking conditions [17].

In this work, different nutrient signaling pathways were analyzed in wine yeasts. The use of chemical inhibitors of these pathways was not useful as predictive tools of the nutritional requirements of commercial *S. cerevisiae* strains, but showed that genetic diversity existed in the activities of such pathways between different industrial isolates. In commercial wine yeasts under standard laboratory conditions, the molecular markers involving the TORC1, Snf1 and NCR pathways basically reacted in the expected ways after nutritional changes and the use of inhibitors. Thus they proved good tools for analyzing the activation of signaling pathways throughout winemaking. During synthetic grape juice fermentation, the activation of TORC1 and NCR was confined to the first fermentation stages, even before growth stopped, while Snf1 was active early in fermentation, despite the repressing amount of sugars. PKA showed activation for both long and short fermentation times. Therefore, grape juice fermentation represents a process with a distinct profile of nutrient signaling pathway activities.

## Results

### Wine strains display intrinsic resistance to rapamycin and 2-deoxyglucose compared to laboratory strains

The first aim of this work was to test the feasibility of using chemicals that target nutrient signaling pathways as probes to screen wine yeast strains for their nutrient requirements. To do so, 14 commercial strains with different nitrogen requirements, according to their manufacturer, were selected (Additional file 1: Table S1). No differences appeared among strains for canavanine sensitivity (Additional file 2A). Next the inhibitor of branched amino acid biosynthesis sulfometuron methyl [18], was tested (Additional file 2B). Some strains with considerable nitrogen needs, such as CKS102 and UCLM S325, displayed slightly increased tolerance, but other strains with minor requirements, such as EC1118 and T73, grew well in the presence of the inhibitor. Next two described TORC1 inhibitors were tested: methionine sulfoximine (MSX; Additional file 2C), and rapamycin (Additional file 2D). No large differences appeared among strains related to MSX. Rapamycin gave wider variability. Related strains DV10 and EC1118 were the most tolerant, but no common pattern was observed in all low-requirement strains, such as 71B and T73. M2 seemed the most sensitive, but this was not shared with other strains that required high nitrogen and showed average growth, such as CY309 or CKS102. Therefore, these inhibitors did not act as good screening tools to rate strains according to nitrogen requirements, but revealed genetic diversity.

**Fig. 1:**
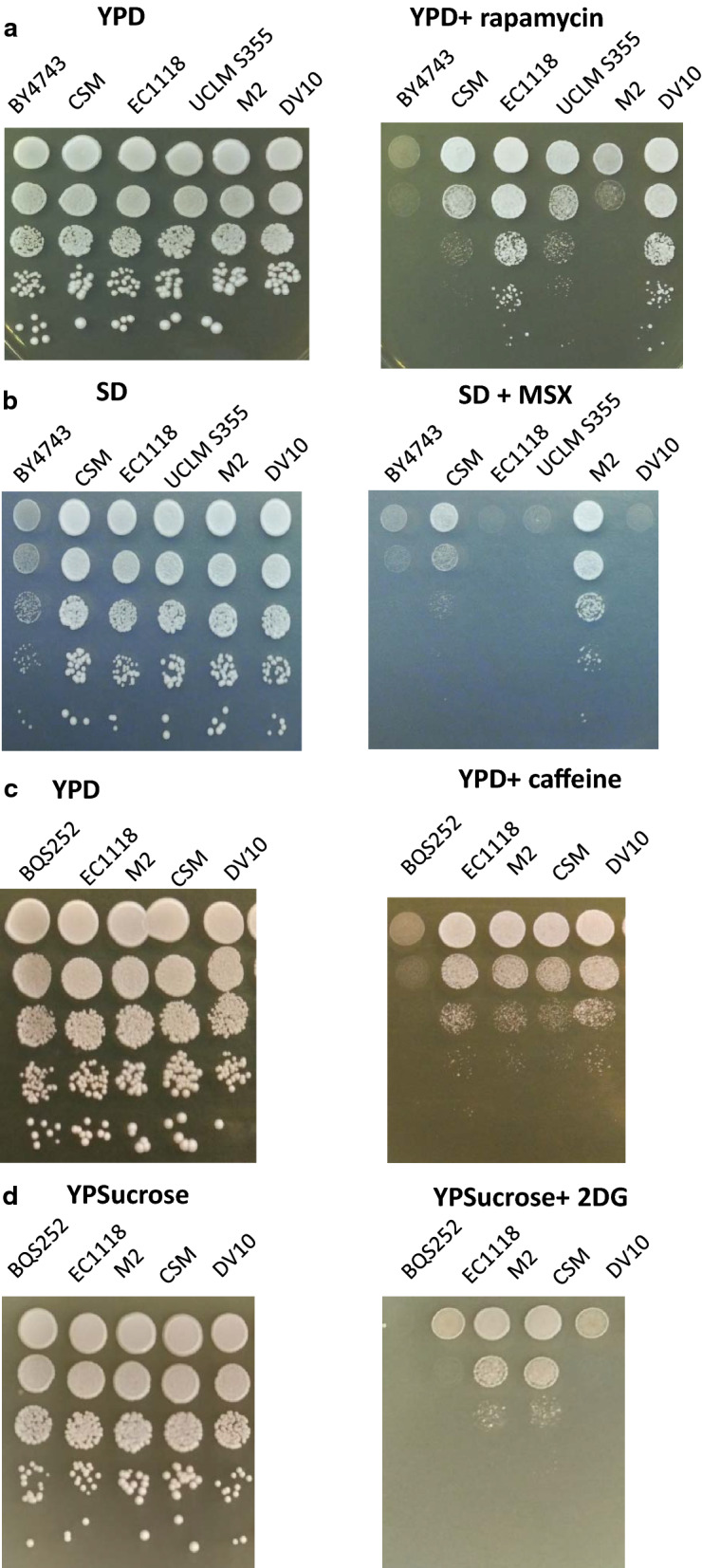
Spot growth analysis of the selected commercial and laboratory strains of *S. cerevisiae*. Serial dilutions were spotted on the plates containing: **a** 100 nM rapamycin in YPD; **b** 1 mM methionine sulfoximine in SD; **c** 1 mM caffeine in YPD; **d** 200 µg/mL of 2-deoxyglucose (2DG) in YPD. Cells were grown at 30 °C for 2 days

The strains to be studied from this point onward were narrowed down according to their sensitivity to rapamycin (Fig. 1a), and the new sets of experiments were restricted to fewer strains given their complexity. Laboratory strains were also tested as an external reference. Figure 1a shows that diploid laboratory strain BY4743 was more sensitive to rapamycin than any commercial wine strain. BQS252, a haploid strain of the same background as BY4743, but with no auxotrophies that affect amino acid synthesis, was used throughout this work (see below). Once again, this strain was more sensitive to rapamycin (data not shown). Strains EC1118 and DV10 were the most tolerant to rapamycin (Fig. 1a). These strains are genetically similar according to their genome sequence [19]. Therefore, the genetic determinants of rapamycin tolerance may be common. M2 was the most sensitive wine strain to rapamycin. CSM and UCLM S355 had average sensitivities. High intracellular superoxide levels have been linked to increased rapamycin tolerance [20]. When superoxide levels were measured it was shown that the more tolerant strains to rapamycin (DV10 and EC1118) displayed higher superoxide levels during exponential growth, while the levels of the more sensitive ones (M2 and CSM) were lower (Additional file 3). The superoxide levels in laboratory strain BQS252 were negligible.

The selected strains’ MSX tolerance was next tested, but in minimal medium to increase its effect (Fig. 1b). Laboratory strain BQS252 was very sensitive. Surprisingly, the most tolerant strains to rapamycin were also sensitive to MSX, while the most tolerant one was M2, and CSM had an intermediate phenotype. Caffeine has been proposed to target TORC1, among other pathways [21]. Wine strains proved more tolerant to caffeine than laboratory strains (BQS252 in this case), but no big differences appeared among them.

The response to carbon sources can be studied by using the glucose analogous 2-deoxyglucose (2DG), which causes glucose repression then blocking growth in sucrose. 2DG completely blocked the growth of the laboratory strain (Fig. 1d), but only partially blocked the growth of all the wine tested strains. Rapamycin-tolerant EC1118 and DV10 were more affected by 2DG than strains CSM and M2.

### Wine strains show an altered phosphorylation pattern for some TORC1 targets, but not for Snf1 or Gln3

**Fig. 2:**
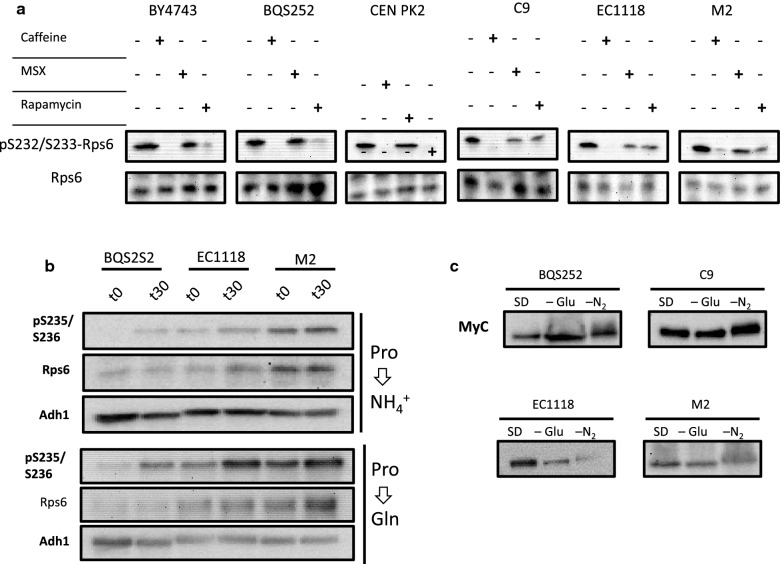
Phosphorylation analysis of the TORC1 targets under laboratory conditions. **a** Immunoblot analysis of Rps6 phosphorylation in the different strains treated with TORC1 inhibitors. The selected laboratory (BY4743, BQS252 and CEN.PK) and industrial strains (C9, EC1118 and M2) were grown in SD medium with the required auxotrophic supplements. Part of the exponentially growing cells was harvested as a control, and the remainder was treated with caffeine (20 mM), MSX (2 mM) and Rapamycin (200 ng/mL) for 30 min. Total lysates were analyzed using the specific anti pS235/S236Rps6 antibody, while total Rps6 was used as the loading control. **b** Rps6 phosphorylation during the transition from a poor nitrogen source (proline) to a good nitrogen source (ammonium or glutamine). The same conditions as in panel **a**. Alcohol dehydrogenase (Adh1) was used as an additional loading control. **c** Par32 phosphorylation during nutrient starvation. Strains carrying the epitope Myc-tagged versions of *PAR32* were changed from SD to minimal medium without either glucose or ammonium sulfate for 20 min

The next step was to study the activation status of the selected nutrient signaling pathways. To do so, the phosphorylation state of well-known targets was analyzed. Ribosomal protein S6 phosphorylation by protein kinase Ypk3 in a TORC1-dependent way using specific antibodies [22, 23] is straightforward TORC1 activity assay that has been tested on wine strains [24, 25]. Laboratory strains BY4743, BQS252 and CEN.PK2 were grown along commercial wine strains EC1118 and M2 and haploid wine strain C9. The cells growing exponentially in minimal medium were treated with two inhibitors that target TORC1, rapamycin and caffeine, which have been described to trigger ribosomal protein Rps6 dephosphorylation [22]. Caffeine caused the expected reduction of Rps6 phosphorylation in all the cells (Fig. 2a). Rapamycin triggered clear dephosphorylation in the laboratory strains, but the intensity of the band corresponding to phosphorylated Rps6 was more marked in all three wine strains, which also indicates altered sensitivity to rapamycin in molecular terms. MSX did not cause dephosphorylation in the laboratory strains, but had a partial effect on strains C9 and EC1118. Therefore, molecular differences appeared in the way that the wine strains sensed and reacted to some inhibitors of TORC1.

Next a change in the quality of the nitrogen source was tested. Cells were grown in proline, a non-preferred nitrogen source, and were then shifted to ammonium, a preferred one (Fig. 2b). In laboratory strain BQS252, the expected behavior was observed: Rps6 phosphoprylation was negligible during growth in a poor nitrogen source, and was visible only after the shift to a rich nitrogen environment by inducing the full TORC1 function [23]. Under the same conditions, the two wine strains (EC1118 and M2) showed an induction in Rps6 phosphorylation, but they already displayed a high degree of phosphorylation under basal conditions. Wine strains also displayed a higher total Rps6 level, so perhaps the signal could be more easily transduced or the degree of phosphorylation could differently affect protein stability. Similar results were obtained when cells were shifted to another rich nitrogen source, namely glutamine (Fig. 2b).

Another branch of the TORC1-controlled network acts on kinase Npr1, that promotes phosphorylation in protein Par32 in poor nitrogen sources [26]. The *PAR32* chromosomal locus was tagged with the Myc (Fig. 2c). Cells were shifted to a medium with no carbon or no nitrogen to follow changes in mobility due to phosphorylation [26]. Glucose depletion brought about no change in electrophoretic mobility in any tested strain, while nitrogen starvation caused Par32 phosphorylation. As similar behavior was seen in laboratory strain BQS252 and also in the three tested wine yeasts, apparently there were no genetic differences in this TORC1 signaling branch.

**Fig. 3:**
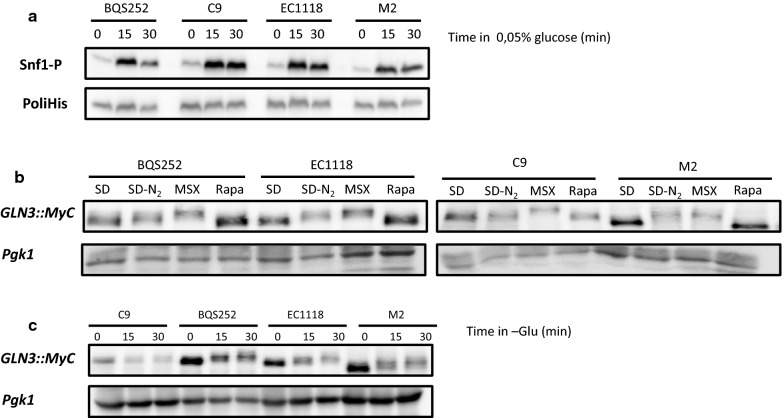
Phosphorylation analysis of Snf1 and Gln3 under laboratory conditions. **a** Immunoblot analysis of the Snf1 phosphorylation in strains BQS252, C9, EC1118 and M2. Cells were grown exponentially in medium with 2% glucose and shifted to derepressing conditions in 0.05% glucose. Proteins were analyzed using the specific anti p-AMPK α (Thr172) antibody, while total Snf1 protein was detected by an anti poly-His antibody. **b** Gln3 phosphorylation during nutrient starvation. The epitope Myc-tagged versions of *GLN3* were changed from SD (0.1% (NH_4_)_2_SO_4_) to minimal medium without ammonium sulfate of 2 mM MSX, or 200 ng/mL of rapamycin were added for 20 min. Glycolytic protein Pgk1 was used as the loading control. **c** Similar experiment as in panel **b**, but by testing glucose removal for 15 and 30 min

The key control protein kinase of glucose repression is Snf1. Phosphorylation at the T210 residue indicates that glucose repression is relieved and the kinase itself is active. The previously tested strains were exponentially grown in 2% glucose and shifted to non-repressing conditions at 0.05% glucose [27, 28] (Fig. 3a). Under these conditions, laboratory strain BQS252 showed the expected behavior: quick Snf1 phosphorylation after only 15 min. This activation then reduced after 30 min, which indicates that the maximum of activation was transient. The same pattern was observed for the three tested wine strains, which indicates that glucose repression basically worked identically in all the *S. cerevisiae* strains.

The GATA transcription Gln3 factor is a key player in NCR, and its phosphorylation status has been linked to both TORC1 and Snf1. *GLN3* was tagged with the Myc epitope and its phosphorylation status was determined by stimuli that control TORC1 activity (Fig. 3b). The cells growing exponentially in minimal medium SD in the presence of ammonium were shifted to a nitrogen-depleted medium. Electrophoretic mobility was lower, which indicated an increased phosphorylation level. Reduced mobility was more evident in commercial strains M2 and EC1118, but was also detected in haploid wine strain C9 and laboratory strain BQS252, albeit to a lesser extent. The shift was more apparent for all the strains when glutamine starvation was caused by MSX. Rapamycin has been described to trigger complete dephosphorylation and the activation of Gln3. For all the strains under this condition, the band displayed the highest mobility in relation to other treatments, but showed no differences in phosphorylation compared to the control growth condition in SD medium. Glucose starvation also caused Gln3 phosphorylation in all the tested laboratory and industrial strains (Fig. 3C). Phosphorylation was apparent after only 15 min of starvation and increased after 30 min. Overall, Gln3 phosphorylation behaved similarly for the wine strains compared to the laboratory reference strain BQS252. Therefore, the analysis of the phosphorylation state of nutrient signaling targets can be used to study the activation of pathways during winemaking.

### TORC1 and Snf1 activity is restricted to the start of fermentation

**Fig. 4:**
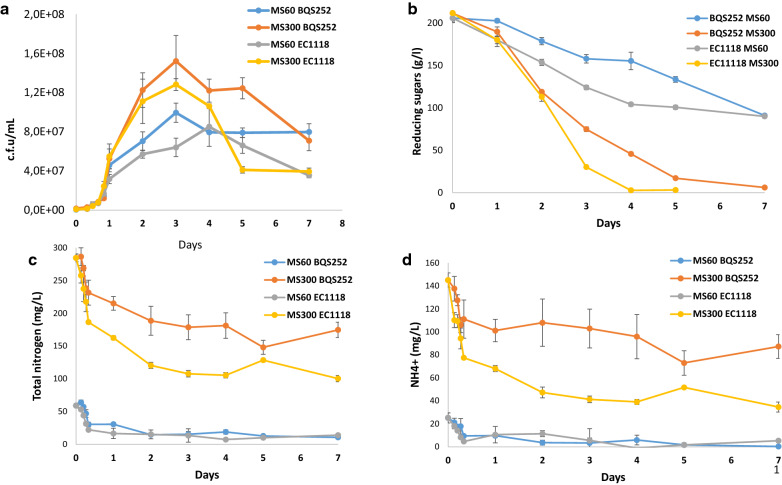
Fermentation profiles of laboratory BQS252 and wine EC1118 strains in synthetic musts MS300 and MS60. **a** Cell growth. Samples were taken during fermentation, diluted and plated, and colony-forming units were counted. **b** Reduced sugar consumption during fermentation. **c** The total assimilable nitrogen content in the growth medium, which resulted from the sum of ammonium and α-amino acids. **d** Ammonium content during grape juice fermentation

For the fermentation experiments, industrial wine strain EC1118 was selected for its commercial relevance, and also because it has been subjected to several transcriptomic and proteomic analyses [14, 29, 30]. Laboratory strain BQS252 was selected because it lacks amino acids auxotrophies. Fermentations were done in synthetic grape juice [31] due to the higher reproducibility and composition control, and also to make easier comparisons to the available global data obtained in the same medium [14, 30]. In fact two synthetic must versions were tested: one with plenty of nitrogen (MS300, 300 mg/l of assimilable nitrogen) and another with restricted nitrogen (MS60, 60 mg/l of assimilable nitrogen). Figure 4 offers, as example, the fermentative behavior of the *PAR32*::Myc versions of both strains, but fermentation proceeded similarly in the wild-type and *GLN3*::Myc strains (data not shown). Low nitrogen caused slower growth and lower maximal cell counts for both strains (Fig. 4a). Growth peaked on day 3 for both strains in MS300 and then viability started to drop, particularly in EC1118, a strain with a short chronological life span [32]. In cell count terms, the laboratory strain fared well compared to the industrial strain, but in fermentative power terms, as measured by sugar consumption, EC1118 was faster than BQS252 (Fig. 4b). As expected, low nitrogen resulted in a much slower sugar consumption rate for both strains. Total nitrogen (sum of ammonium and amino acids) was depleted mostly from limiting medium MS60 during the first fermentation hours (Fig. 4c). In the plentiful MS300, nitrogen was very quickly uptaken on day 1, and EC1118 consumed around half the total nitrogen before lowering the consumption rate. However, nitrogen was not completely consumed, which meant that it was not really limiting under this condition. The wine strain showed better nitrogen assimilation than the laboratory strain, which probably gave it a performance edge. This difference was due mostly to its better ammonium uptake (Fig. 4d).

**Fig. 5:**
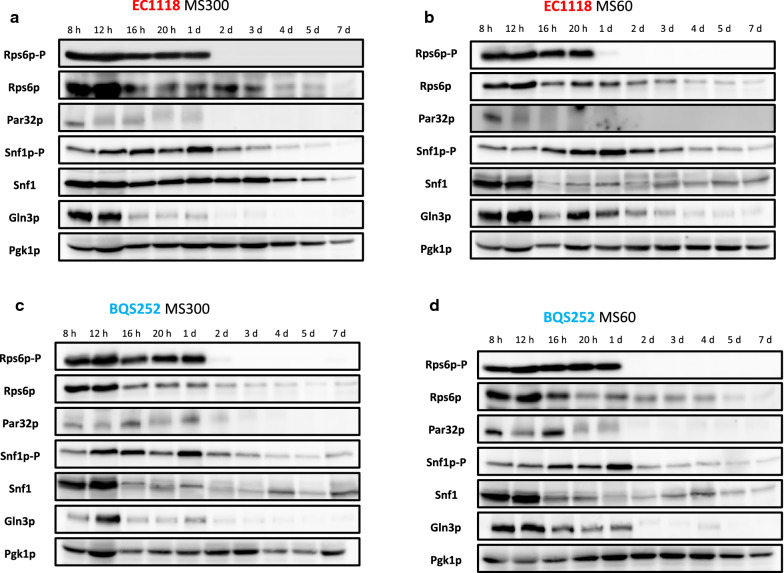
Phosphorylation of the nutrient signaling markers during fermentation in synthetic musts. Strains EC1118 (**a**, **b**) and BQS252 (**c**, **d**) were grown in synthetic must MS300 (**a**, **c**) and MS60 (**b**, **d**). Specific antibodies against the Rps6 and Snf1 phosphorylated forms were used. A specific antibody against Rps6 was employed as the loading control for Rps6 phosphorylation and the anti-polyHis antibody was utilized to detect total Snf1. An anti-Myc antibody was employed to detect the epitope tagged versions of Par32 and Gln3. Pgk1 was used as the loading control

**Fig. 6:**
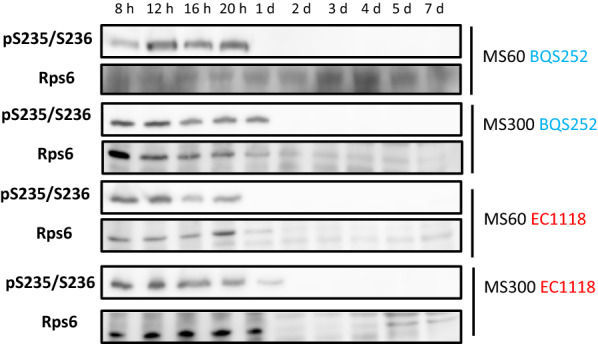
Western blot analysis of Rps6 phosphorylation along fermentation using an alternative protein extraction method. Strains EC1118 and BQS252 were grown in synthetic grape juice MS300 and MS60 and cellular extracts were obtained by mechanical breakage using glass beads. Specific antibodies against Rps6 phosphorylated and total forms were used

The phosphorylation status of target proteins at the different time points during fermentation was analyzed in strain EC1118 or in the corresponding Myc-tagged versions for Par32 and Gln3, where tagging did not impair fermentation (data not shown) (Fig. 5). Samples were taken at 8 h, 12 h, 16 h, 20 h and on days 1, 2, 3, 4, 5 and 7. After these time, cells were too old and the protein analysis became messy. First, Rps6 phosphorylation was followed as a marker of TORC1 activation. Under the reference plenty nitrogen condition (MS300; Fig. 5a), a clear band was detected until day 1 with no apparent differences appearing. Then the phosphorylated protein completely disappeared on day 2, which indicates that this branch of TORC1 was active only during the very first fermentation hours, and ceased even before growth stopped. This phosphorylation disappeared as total Rps6 was still detected on days 2 and 3 at similar levels to those recorded on day 1. Later it became very faint on days 4 and 5, and was undetectable after 1 week, probably due to a general decrease in the total protein extracted in the stationary phase (see loading control protein Pgk1). When nitrogen was reduced, MS60 (Fig. 5b), the pattern was similar, but the phosphorylated Rps6 band was no longer detected after 20 h, but there was a detectable amount of total Rps6 at that point and later. Therefore, the initial TORC1 activity burst occurred during the first hours and its span reflected the amount of nitrogen in the medium. The Western blots showed in the figure display the proteins extracted with TCA (see Materials and Methods), a fast method required to see transient Snf1 phosphorylation and to prevent Gln3 degradation (see below). The results obtained by an alternative protein extraction method involving the mechanical breaking of cells with glass beads and crude extract clarification by centrifugation gave similar results (Fig. 6). Overall, similar results were obtained for laboratory strain BQS252 despite their different genetic backgrounds (Fig. 5c, d). In this case, behavior was similar under low and high nitrogen conditions, with only phosphorylation present on the first day, which suggests an attenuated mechanism for sensing nitrogen despite the fermentation in both synthetic grape juices being quite different.

Next another target regulated by TORC1 in response to nitrogen starvation, Par32, was analyzed (Fig. 5). As seen in Fig. 2c, nitrogen starvation led to increased Par32 phosphorylation. Wine strain EC1118 in MS300 showed a band at 8 h that started an increase in shifting to reach the minimal electrophoretic mobility at 20 h before its migration increased on day 1 and then disappeared after 2 days (Fig. 5). The pattern was similar in MS60, with apparent nitrogen starvation at only 20 h (Fig. 5b). However after 1 day plenty of nitrogen was left in the fermentations carried out in rich MS300 (see Fig. 4c), which suggests no proportional phosphorylation response in Par32. The protein disappeared in both environments after 2 days. The kinetics of both Par32 phosphorylation and degradation were similar in BQS252, but the band was still visible on day 2 of MS300 fermentation. This scenario suggests that the process slowed down in the laboratory strain, possibly because it was not capable of quick adaptation in the winemaking environment.

**Fig. 7:**
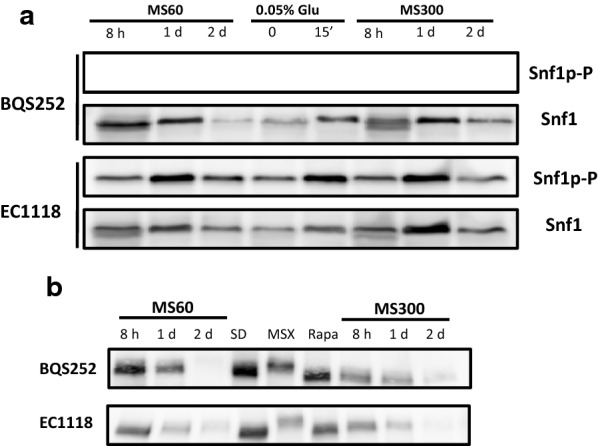
Direct comparison made between the laboratory and winemaking conditions. **a** Snf1 phosphorylation was measured by a Western blot analysis. The samples from strains EC1118 and BQS252 under laboratory conditions of derepression at a low glucose concentration, as described in Fig. 3a, were run together with the selected samples (8 h and days 1 and 2) under winemaking conditions (Fig. 5a). A specific antibody against the Snf1 phosphorylated form was used. An anti-polyHis antibody was utilized to detect total Snf1. **b** The Gln3 phosphorylation Western blot analysis. The samples from strains EC1118 and BQS252 under laboratory conditions with the addition of MSX and rapamycin, as described in Fig. 3b, were run together with the selected samples (8 h and days 1 and 2) under winemaking conditions (Fig. 5a). The tagged version of Gln3 was detected with an anti-Myc antibody

Snf1 kinase activity may provide information on the overall carbon source metabolism. Strain EC1118 showed Snf1 phosphorylation in MS300 at the very first time point (Fig. 5a) and the intensity of the band increased to peak on day 1 of growth. Then it diminished and became almost undetectable on day 5. The total amount of Snf1 remained higher for a longer time, and was fairly constant up to day 3 before reducing. The pattern in low nitrogen MS60 was similar, with an increase noted until day 1 before lowering, which was less dramatic in this case, and phosphorylated Snf1 was detectable until the last time point (Fig. 5b). However, total Snf1 declined more sharply after the first two time points in MS60, and became consistently lower afterward throughout fermentation. BQS252 had a similar profile, with activation peaking on day 1 for both MS300 and MS60 (Fig. 5c, d). The total Snf1 protein had a similar profile to EC1118 in MS60, with a larger amount of protein up to 12 h before suddenly falling to a constant level. In any case, Snf1 did not show the expected phosphorylation pattern, which is consistent with activation at low glucose concentrations as sugar content was very high upon its activation peak (see Fig. 4b). To assess the degree of Snf1 activation, the samples selected from the fermentation experiments were loaded together with the samples from cultures for high (2%) and low (0.05%) glucose under laboratory conditions (as seen in Figs. 2, 7a). In both strains and both synthetic musts, the highest degree of Snf1 phosphorylation on day 1 gave a similar intensity to the derepressed sample. Therefore, Snf1 was activated at the very start of wine fermentations by probably reacting to stressful conditions or other physiological imbalances rather than to sugar scarcity.

For Gln3, a strong signal was observed in strain EC1118 growing in MS300 for the first 12 h, a fainter amount of protein was noted until day 1, but even less was observed after that time point (Fig. 5a). The nitrogen scarcity in MS60 was sensed by the NCR system as Gln3 levels were higher for longer times, although its maximum was still noted on the first day of growth (Fig. 5b). The BQS252 pattern was similar, particularly in MS300. In this case, the amount of Gln3 clearly peaked at 12 h (Fig. 5c). In MS60, very little Gln3 was found after day 1 (Fig. 5d). A slight shift in band size seemed to occur at longer times. So in order to see this in detail, selected samples from fermentation were run with the samples from Gln3 in the laboratory condition experiments shown in Figs. 2, (7b). The samples of EC1118 at 8 h in MS300 showed slightly lower mobility than the basal rapamycin-treated samples, similarly to the exponential growth in SD (Fig. 7b, lower panel). Mobility slightly diminished on day 1, but far from the MSX-treated samples. A similar profile occurred in MS60, with a decrease up until day 2. This was consistent with an initial peak of Gln3 activity that dropped after its phosphorylation. In BQS252, the phosphorylation levels seemed low in MS300, similarly to the rapamycin-treated sample, while the migrations in MS60 were similar to the samples grown in SD (Fig. 7b, upper panel). These results reinforce the idea that Gln3 is active, at least for the first hours of vinification, thus NCR does not seem fully in place under these conditions.

### PKA acts throughout fermentation

**Fig. 8:**
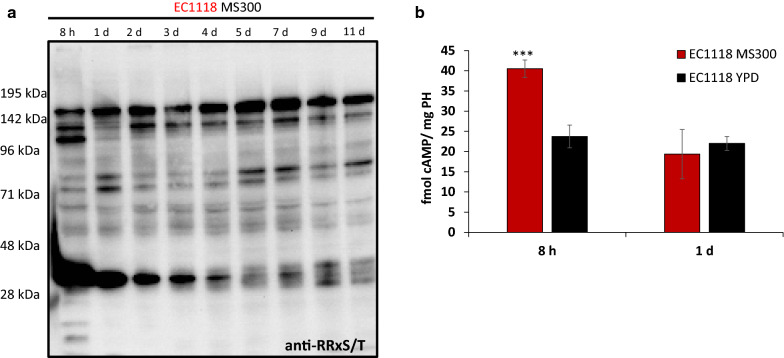
Analysis of PKA activity. **a** Pattern of the proteins phosphorylated by PKA by a Western blot analysis using an anti-phosphorylated PKA consensus RRXS/T in strain EC1118 grown in synthetic must MS300. **b** cAMP quantification from the extracts of the same strain grown in MS300 for 8 h and 1 day, and in YPD medium in both the exponential and stationary phases

Next step was to analyze the remaining main nutrient signaling pathway, PKA. The samples from a standard fermentation in MS300 by EC1118 were tested for PKA activity by using a specific antibody against the phosphorylated PKA consensus, RRxS/T (Fig. 8a). The experiment showed a dynamic complex pattern of multiple targets whose relative abundance changed with time. Some bands appeared at the very first time point of 8 h before lowering on day 1. Some remained fairly constant throughout fermentation, while others appeared only after longer times. These results suggest that the PKA pathway remained activated throughout fermentation. Considering that it responds to the presence of fermentable sugars, it makes sense that it is active while fermentation is underway. In an attempt to identify any of the detected bands, the deletion of the genes coding for the known PKA targets (*CKI1*, *MAF1*, *LHP1*, *ATG18* and *YPK3* [33, 34]) was performed in haploid wine strain C9 (Additional file 4). None of these deletions caused any main band with the expected size to disappear (Additional file 4 and data not shown). Hence we were unable to identify any of the main PKA targets during winemaking, which may seem to differ from those under laboratory conditions. In order to estimate the activity of the adenylate cyclase, cAMP levels were measured for strain EC1118 at 8 h and 1 day of fermentation, and at the exponential and stationary growths in rich YPD medium (Fig. 8b). Levels were higher after 8 h of growth in MS300 than on day 1. Therefore, an initial burst of activity seemed to be confirmed and to promote growth. Later, the cAMP level was similar to those observed under laboratory conditions, which suggests proper PKA activation under winemaking conditions.

## Discussion

Nutrient-sensing and signaling pathways are key factors for orchestrating the complex physiological mechanisms underlying the adaptation of microorganisms to changing environments during their biotechnological use. The coordination of different pathways that react distinctly to each nutrient is key to control growth, metabolism and stress responses [4]. *Saccharomyces cerevisiae,* which is used for winemaking, is no different in this respect. Nitrogen deficiency in grape juice is one of the main technological problems that industry faces [1]. Nitrogen scarcity and other nutritional imbalances can lead to stuck and sluggish fermentations that are very costly for the industry [12]. This is why attention has been paid to nitrogen-related pathways (particularly NCR) when wine yeast physiology during fermentation has been studied. However, very little information is available on the integration of different pathways, or about the behavior of distinct industrial isolates in nutrient-sensing mechanisms. Chemical screening was performed on 14 commercial strains with different nitrogen requirements by using a series of inhibitors of amino acid biosynthesis and toxic analogs of amino acids and glucose. However, none proved useful as a diagnostic tool to discriminate between low and high nutrient requirements, but indicated that genetic variability existed between commercial strains and could be exploited to identify suitable new ones. For instance, strains EC1118 and DV10 have been described as being genetically related, both belong to the Prise de Mousse clade [19]. They possess very similar tolerance to the tested chemicals (Fig. 1) with, for instance, the best tolerance to rapamycin and the worst to 2-deoxyglucose, and both have low nitrogen requirements. These data could be used to identify strains displaying similar behaviors.

Clearer differences appeared when comparing the laboratory strains to the wine strains as the latter were more tolerant to rapamycin, caffeine and 2-deoxyglucose (Fig. 1). The altered carbon catabolite repression in the industrial yeasts (indicated herein by higher tolerance to 2-DG) has been previously reported as they consumed sucrose first, even in the presence of glucose [35]. This behavior is not due merely to altered invertase activity because the same tolerance to 2DG was observed when glycerol was used as a carbon source (data not shown). Glucose repression ceases by Snf1 protein kinase activation when glucose drops below a certain threshold. The profile of Snf1 phosphorylation, which leads to its activation after changing to derepressing conditions, is similar between laboratory and wine strains (Fig. 3a), with peak activation occurring 15 min after incubation at low sugar concentrations. Thus the upstream part of the pathway, which senses the presence or absence of glucose, seems to work similarly. The signal seemed stronger in the wine strains after 30 min at low sugar concentrations, which suggests greater long-term activation that could provide growth in a non-repressing situation. Further work is necessary to find the source of the molecular differences between these strains, which may be related to either the transcription factors imposing glucose repression (e.g. Mig1 and Adr1) or the phosphatases acting on Snf1. Wine strains are more tolerant to two TORC1 inhibitors, rapamycin and caffeine, which suggests greater TORC1 activity. These inhibitors have different targets in the complex, Fpr1 for rapamycin and Tor1 kinase for caffeine [36], which indicates that chemical tolerance is not due to alterations in the binding sites of these components. Molecular differences may reside downstream in one of the many direct or indirect TORC1 targets that TORC1 can alter either direct or indirectly. When known markers of TORC1 activity were tested against inhibitors under laboratory conditions to compare both sets of strains (Figs. 2, 3), some targets, like Par32 and Gln3, showed no striking differences, while ribosomal Rps6 phosphorylation displayed differences when cells were treated with rapamycin, with Rsp6 phosphorylation exhibiting slightly more persistence. As this was not the case for caffeine, differences may occur in the Fpr1 subunits of the TORC1 complex, the target of rapamycin, although genomic sequences do not show variability in the *FPR1* gene (data not shown). Phenotypic variability in TORC1 activation has been shown for the main phylogenetic *S. cerevisiae* lineages [37], so these differences may reflect adaptation to different environments. Therefore, minor differences in the branches of the pathways may lead to differences in growth in the presence of such inhibitors, but further work has to be done to identify the genetic differences in wine strains that may lead to such phenotypes, and to also evaluate the relevance for their industrial role.

As most analyzed reporter proteins responded to nutrient starvation and chemical inhibitors as expected, they can be used to track the status of different pathways during synthetic grape juice fermentation (Figs. 5, 6, 7). The results obtained in the laboratory strain generally matched those observed for wine strain EC1118 (Fig. 5), so we can assume that the molecular mechanisms described in the literature for model yeast strains apply to this different environment. Active TORC1 is expected to be observed at the beginning of fermentation, when nutrients are plenty to promote growth. Rps6 phosphorylation indeed indicated that this was the case for the first hours. When nitrogen was poor in MS60, its activity only lasted 20 h, and this matched the consumption of most nitrogen (Fig. 4). However when nitrogen was plenty in MS300, and neither ammonium was, nor amino acids, were exhausted after 1 day, Rps6 was no longer phosphorylated. This means that TORC1 inhibition did not correlate with nitrogen availability. Par32 phosphorylation also indicated TORC1 inhibition, which started in MS300 after only 12 h and would relieve ammonium transporter activity [26]. Clearly no shortage of ammonium in the media after that time in MS300 took place, which could indicate that yeasts are programmed to consume amino acids over ammonium in very early stages. However, TORC1 did not sense amino acids outside the plasma membrane. The complex is situated in the vacuolar membrane, and the important factor for signal transduction is the intracellular balance of nitrogen compounds because some amino acids, e.g. glutamine and leucine, impact TORC1 more strongly than others. In any case, clearly TORC1 activity initially burst, which ceased before cellular growth stopped (Fig. 4). Thus it would seem that, from the very beginning, the molecular mechanisms dependent on TORC1 are set and can continue without requiring the active complex to keep the signal strong.

The Snf1 protein kinase case was even more striking as phosphorylation and activation were expected to occur upon sugar depletion. However, phosphorylation peaked and then remained active after only 1 day, when sugars were plenty and fermentation was very active. The stress activation of Snf1 probably occurs, as it has been described for several adverse conditions, such as high pH, or salt and oxidative stress, but not for sorbitol and heat shock stress [38]. These individual stresses did not match the usual adverse conditions in early and mid-fermentations at a low pH and high osmotic pressure, but the overlap of stressing factors could have a similar effect on Snf1 activation. Global transcriptomic analyses during vinification have detected an unusually high expression of some genes subjected to glucose repression (including *SUC2*, which codes for invertase), but this does not occur for most [14, 39]. Therefore, Snf1 activity during fermentation is consistent with the observation that some of its targets may be expressed during wine making, but it would not imply full derepression as indicated by, for instance, the low expression of respiratory genes through fermentation [14].

Gln3 is one of the GATA transcription factors whose activity is required to alleviate NCR. This process is also controlled in an antagonist manner by TORC1. Nonetheless, some NCR‐sensitive gene transcription can be triggered in the presence of active TORC1 [40], which means that alternative pathways operate to control nitrogen metabolism. Gln3 is also phosphorylated, and possibly inactivated, when glucose concentrations are low (Fig. 3), and it has been described as a target of Snf1 kinase. Hence all the analyzed pathways could converge on this transcription factor. The expected Gln3 pattern would be its accumulation at later fermentation times, when preferred nitrogen sources were depleted, but Gln3 content was surprisingly higher at the very beginning of fermentation in both synthetic musts, which were rich and poor in nitrogen (Fig. 5). Lowering nitrogen extends the presence of Gln3, so its expression adapts to nutritional requirements. After the first 12 h, Gln3 phosphorylation and inactivation increased (Figs. 5, 6) to match Par32 and Snf1 phosphorylation. Hence Gln3 inactivation could be due to either of the two pathways, although TORC1 probably plays the leading role in Gln3 activity control. The transcriptomic analysis indicated that the NCR-sensitive genes were strongly expressed upon the onset of nitrogen starvation during fermentation [14]. So it would seem that the activation status of Gln3 preceded that increased expression. The expression of the main amino acid transporter, Gap1, which is subject to NCR, was high during the first fermentation hours when nitrogen was low [13]. Thus Gln3 would be active at the very beginning of winemaking despite other factors. However, the global transcriptomic analyses indicated that the genes repressed by NCR defined under laboratory conditions were not expressed in a coordinate manner under winemaking conditions [15]. Therefore, exhaustive work has to be done to understand the Gln3 function and its targets in winemaking because it clearly receives a completely different combination of stimuli. The C/N ratio is a key factor for controlling cell physiology. We determined that a high carbon/low nitrogen ratio of musts would influence the impact of TORC1 on longevity [17]. Similarly, the unusual ratio of both nutrients, compared to laboratory media, could involve the unexpected activation of some proteins, like Snf1 or Gln3, through mechanisms that are yet to be described.

Further work is required to understand how these pathways work together under winemaking conditions and what the hierarchy among them is. According to the literature and our results, it seems plausible that PKA works above them all, where the phosphorylation of many targets changes differently through fermentation (Fig. 8). Although an initial peak in the cAMP concentration took place during fermentation (Fig. 8b), PKA was probably active as long as sugars were available, i.e. during most fermentation. This would set a framework within which the other signaling pathways would fluctuate according to other nutrient changes and requirements.

## Conclusions

The use of chemical inhibitors targeting nutrient signaling pathways reveals genetic heterogeneity among commercial wine strains. However, all industrial strains are more tolerant to rapamycin and 2-deoxyglucose than laboratory strains, and this suggests a common adaptation to certain environmental conditions. In molecular terms, nutrient signaling pathways in wine yeasts react to the change in nitrogen and carbon sources, and mostly in the expected way. However, under winemaking conditions, the activation of Snf1 and Gln3 takes place early and does not imply sugar or nitrogen starvation. TORC1 activity is restricted to the first hours of fermentation, and it stops before nitrogen is exhausted and growth stops, showing an unexpected behavior compared to laboratory conditions. PKA is the only pathway that is active at late fermentation and that may indicate that it controls the rest of the molecular events. What all this indicates is that grape juice fermentation involves complex metabolic conditions which trigger a novel response in molecular terms.

## Methods

### Yeast strains, growth media and conditions

The yeast strains herein used are listed in Additional file 1. Plasmid pFA6a-13Myc-kanMX6 [41] was used as a PCR template for the Ct Myc-epitope tagging of *GLN3* and *PAR32* in strains EC1118 and BQS252. Yeast transformation was carried out by the lithium acetate method [42]. For maintenance and daily propagation purposes, yeasts were grown at 30 °C in rich YPD medium (1% yeast extract, 2% bactopeptone, 2% glucose). Minimal medium SD contained a 17% yeast nitrogen base, 0.1% or 0.5% ammonium sulfate, and 2% glucose. Solid media contained 2% bacteriological agar, and 200 mg/l geneticin whenever required to select transformants. Synthetic grape juice MS300 was made as previously described (33). MS60 was made by proportionally reducing the amount of amino acids and ammonium.

For tolerance to chemicals, the stationary phase cultures in YPD were subjected to a series of tenfold dilutions, and 5 μl of each dilution were spotted on the plates containing the indicated amount of inhibitor on either YPD or SD plates and grown at 30 °C for 2 days. To perform the protein phosphorylation analysis in laboratory media, precultures in YPD were used to dilute cells to OD_600_ = 0.1 in the appropriate medium (YPD or SD) and to grow cells to the exponential phase until the required chemical compound was added or the shift to alternative medium took place. For the fermentation experiments conducted in synthetic musts MS300 and MS60, the cells from the stationary cultures in YPD were inoculated at a final concentration of 10^6^ cells/mL in filled-in conical centrifuge tubes with 30 mL of must. Incubation was done with little shaking at 25 °C. The vinification progress was followed by determining colony-forming units (by serial dilution, plating and colony counts) and sugar consumption by reaction to DNS (dinitro-3,5-salycilic acid) (34). α amino acids were measured by OPA/N-acetyl-l-cysteine [43]. Ammonium was analyzed by fluorescence according to the MAK310 ammonium assay kit (Sigma-Aldrich).

### Western blotting

Proteins were extracted by a method that implies fast cell lysis with tricholoacetic acid (TCA) [28, 44]. To 5 OD_600_ units of cells, 5.5% TCA was added and incubated on ice for 15 min before centrifuging cells. The pellet was washed twice with acetone, air-dried and frozen at − 80 °C. Cells were resuspended in 150 μL of 10 mM Tris–HCl, pH 7.5, 1 mM EDTA and broken with 150 μL of 0.2 M NaOH. Cells were centrifuged and the pellet resuspended in gel loading buffer for SDS-PAGE. Alternatively, cells were broken with glass beads and whole cell extracts were extracted in lysis buffer (Tris–HCl 0.1 M, pH 7.5, NaCl 0.5 M, MgCl_2_ 0.1 M, NP40 1% (v/v), PMSF 10 mM and protease inhibitors and phosphatase inhibitors) in a FastPrep-24 (MPBio) shaker for three cycles of 20 s at a speed of 4.5 m/s [45]. Extracts were clarified by centrifugation before being diluted after quantification by the Bradford method (Biorad Inc. Hercules, CA, USA) in loading buffer. Samples were boiled for 5 min before loading. SDS-PAGE was carried out in an Invitrogen mini-gel device. Afterward, the gel was blotted onto PVDF membranes for the immunodetection analysis with a Novex semy dry blotter (Invitrogen, Carlsbad, CA, USA). The used antibodies are described in Additional file 5. The ECL Western blotting detection system (GE) was used following the manufacturer´s instructions.

### cAMP and superoxide determination

Kit RPN2251 (GE Healthcare) was used to measure cAMP with some modifications [46]: 15 OD_600_ of cells were pelleted and frozen in liquid nitrogen. The pellet was washed with PBS and weight was determined. Then 350 µL of Lysis Reagent 2B and 150 µL of glass beads were added. Cells were broken in a FastPrep-24 (MPBio) 3 times for 20 s at a speed of 4.5 m/s. Centrifugation was run for 10 min at 4 °C and 12,000 rpm, and the supernatant was used for the assays by following the nonacetylation protocol described in the kit. Samples were analyzed in triplicate. Superoxide levels were measured by incubating cells in 200 μL PBS with 10 μg/mL of DHE (dihydroethidium) for 15 min at 30 °C and measuring at 485/595 nm in a Varioskan Lux (ThermoFisher) plate reader [47].

## Supplementary information

**Additional file 1.** Strains used in this work. Strains are listed alphabetically, indicating their full commercial name in the case of industrial strains, or genotype in the case of laboratory strains. The nitrogen needs are indicated according to the technical datasheets provided by the manufacturer in their web site (lallemandwine.com and fermentins.com). The precedence or producer of each strain is indicated.

**Additional file 2**. Spot analysis of 14 commercial *S. cerevisiae strains*. Serial dilutions were spotted on plates containing A) 60 mg/l canavanine on SD B) 1 mg/l sulfometuron methyl in SD C) 1 mM methionine sulfoximine in YPD D) 100 nM rapamycin in YPD.

**Additional file 3**. Superoxide levels measured by dihydroethidium (DHE) incubation. Cells of selected strains were grown in YPD and tested in exponential and stationary condition.

**Additional file 4**. Western blot analysis of PKA targets during MS300 fermentation for one day. Haploid C9 strains and mutants in potential PKA targets were used. Membrane was probed with an anti-PKA phosphorylation consensus RRXT/S.

**Additional file 5.** Antibodies used in this work.

## Data Availability

Data sharing is not applicable to this article as no datasets were generated or analysed during the current study.
