# Supplementary material for: Saccharomyces cerevisiae nutrient signaling pathways show an unexpected early activation pattern during winemaking
Source: Microb Cell Fact. 2020 Jun 6;19:124. doi: 10.1186/s12934-020-01381-6 (PMC7275465; doi:10.1186/s12934-020-01381-6)
Supplement: Supplementary file 1 — Additional file 1. Strains used in this work. Strains are listed alphabetically, indicating their full commercial name in the case of industrial strains, or genotype in the case of laboratory strains. The nitrogen needs are indicated according to the technical datasheets provided by the manufacturer in their web site (lallemandwine.com and fermentins.com). The precedence or producer of each strain is indicated. [file 12934_2020_1381_MOESM1_ESM.pdf]

**Additional File 1. Strains used in this work.**

Strains are listed alphabetically, indicating their full commercial name in the case of industrial strains, or genotype in the case of laboratory strains. The nitrogen needs are indicated according to the technical datasheets provided by the manufacturer in their web site (lallemandwine.com and fermentins.com). The precedence or producer of each strain is indicated.

| Strain | Genotype/commercial name | Relative nitrogen needs | Origin |
|---|---|---|---|
| 71B | Lalvin 71B | Low | Lallemand |
| *BC S103* | *SafŒno™ BC S103* | Low | Fermentis |
| BM45 | Lalvin BM45 | Medium-High | Lallemand |
| BQS252 | MAT a *ura3-52*. | N.D. | J. Enrique Pérez-Ortin |
| BQS252 *PAR32*::Myc | BQS252 *PAR32*::13Myc-KanMX6 | N.D. | This work |
| BQS252*GLN3*::Myc | BQS252 *GLN3*::13Myc-KanMX6 | N.D. | This work |
| BY4743 | *MAT**a**/**α** his3*Δ1/*his3*Δ1 l*eu2*Δ0/l*eu2*Δ0 *LYS2*/*lys2*Δ0 *met15*Δ0/*MET15* *ura3*Δ0/*ura3*Δ0 | N.D. | Brachmann et al., 1998 |
| C9 | *Mat**a** ho::loxP* | N.D. | Walker *et al.*, 2003 |
| *CK S102* | *SafŒno™ CK S102* | High | Fermentis |
| CSM | Enoferm CSM | Medium | Lallemand |
| CY3079 | Lalvin CY3079 | Medium-High | Lallemand |
| DV10 | Lalvin DV10 | Low | Lallemand |
| EC1118 | Lalvin EC1118 | Low | Lallemand |
| EC1118 *PAR32*::Myc | EC1118 *PAR32*::13Myc-KanMX6 | N.D. | This work |
| EC1118 *GLN3*::Myc | EC1118 *GLN3*::13Myc-KanMX6 | N.D. | This work |
| L2056 | Lalvin Rhône 2056 | Medium | Lallemand |
| M2 | *Enoferm M2* | Medium-High | Lellemand |
| RC212 | Lalvin RC212 | Medium | Lallemand |
| T73 | Lalvin T73 | Low | Lallemand |
| *UCLM S377* | *SafŒno™ UCLM S377* | High | Fermentis |
| UCLM S325 | SafŒno™ UCLM S325 | High | Fermentis |
