# Supplementary material for: Saccharomyces cerevisiae nutrient signaling pathways show an unexpected early activation pattern during winemaking
Source: Microb Cell Fact. 2020 Jun 6;19:124. doi: 10.1186/s12934-020-01381-6 (PMC7275465; doi:10.1186/s12934-020-01381-6)
Supplement: Supplementary file 2 — Additional file 2. Spot analysis of 14 commercial S. cerevisiae strains. Serial dilutions were spotted on plates containing A) 60 mg/l canavanine on SD B) 1 mg/l sulfometuron methyl in SD C) 1 mM methionine sulfoximine in YPD D) 100 nM rapamycin in YPD. [file 12934_2020_1381_MOESM2_ESM.pdf]

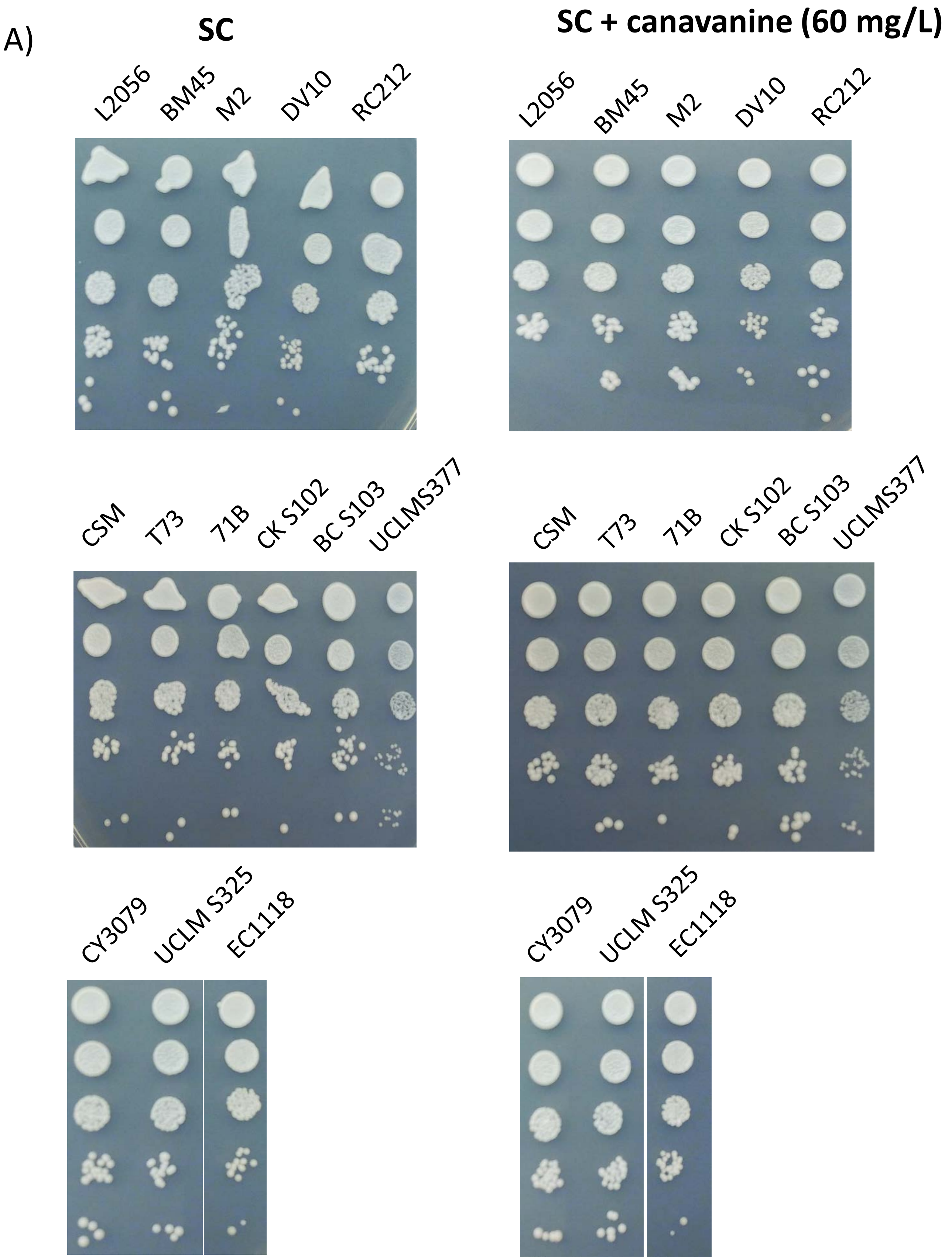
A)
SC
SC + canavanine (60 mg/L)
L2056
BM45
M2
DV10
RC212
CSM
T73
71B
CK S102
BC S103
UCLMS377
CY3079
UCLM S325
EC1118

B)

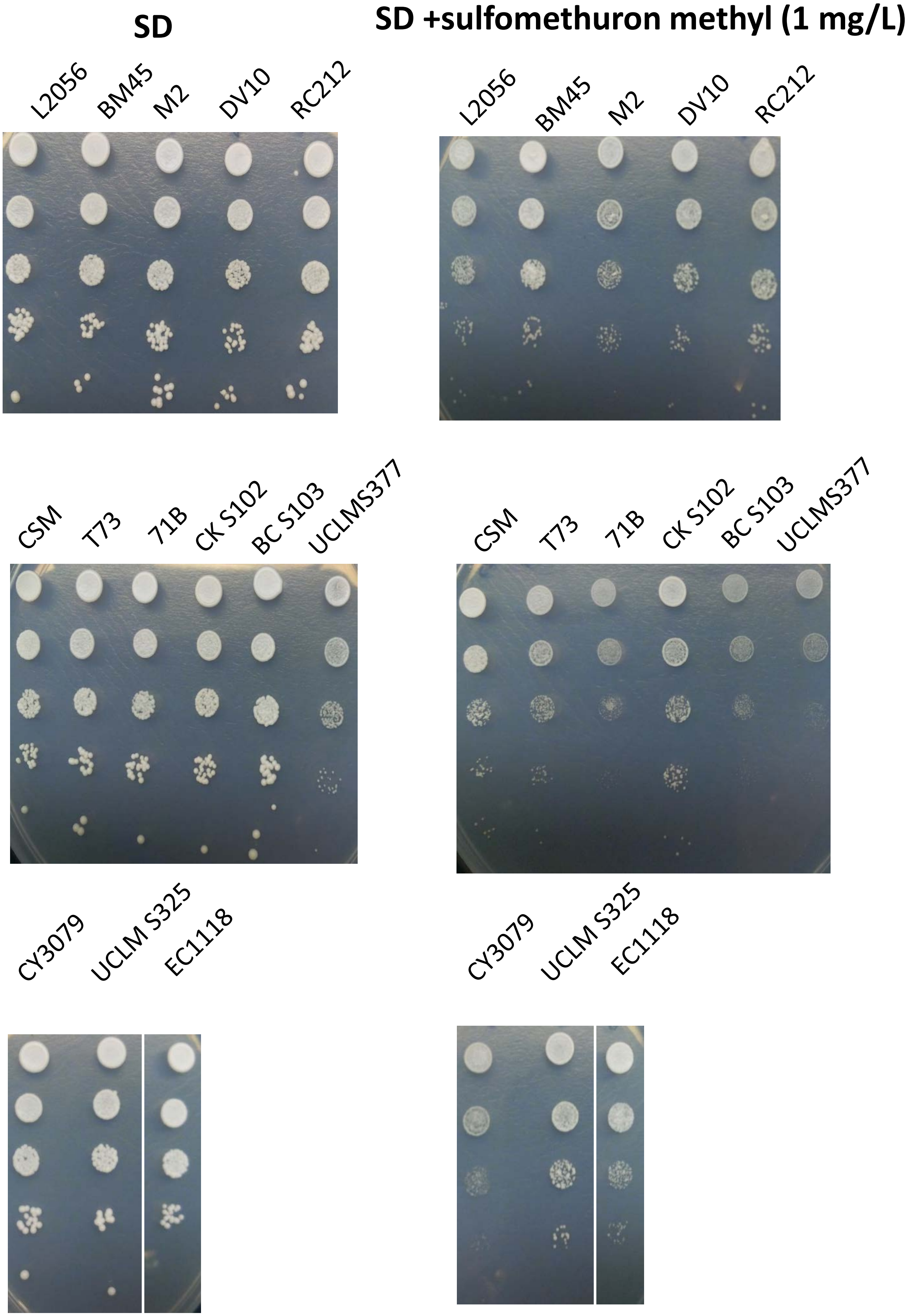
SD
SD +sulfomethuron methyl (1 mg/L)
L2056
BM45
M2
DV10
RC212
L2056
BM45
M2
DV10
RC212
CSM
T73
71B
CK S102
BC S103
UCLMS377
CSM
T73
71B
CK S102
BC S103
UCLMS377
CY3079
UCLM S325
EC1118
CY3079
UCLM S325
EC1118

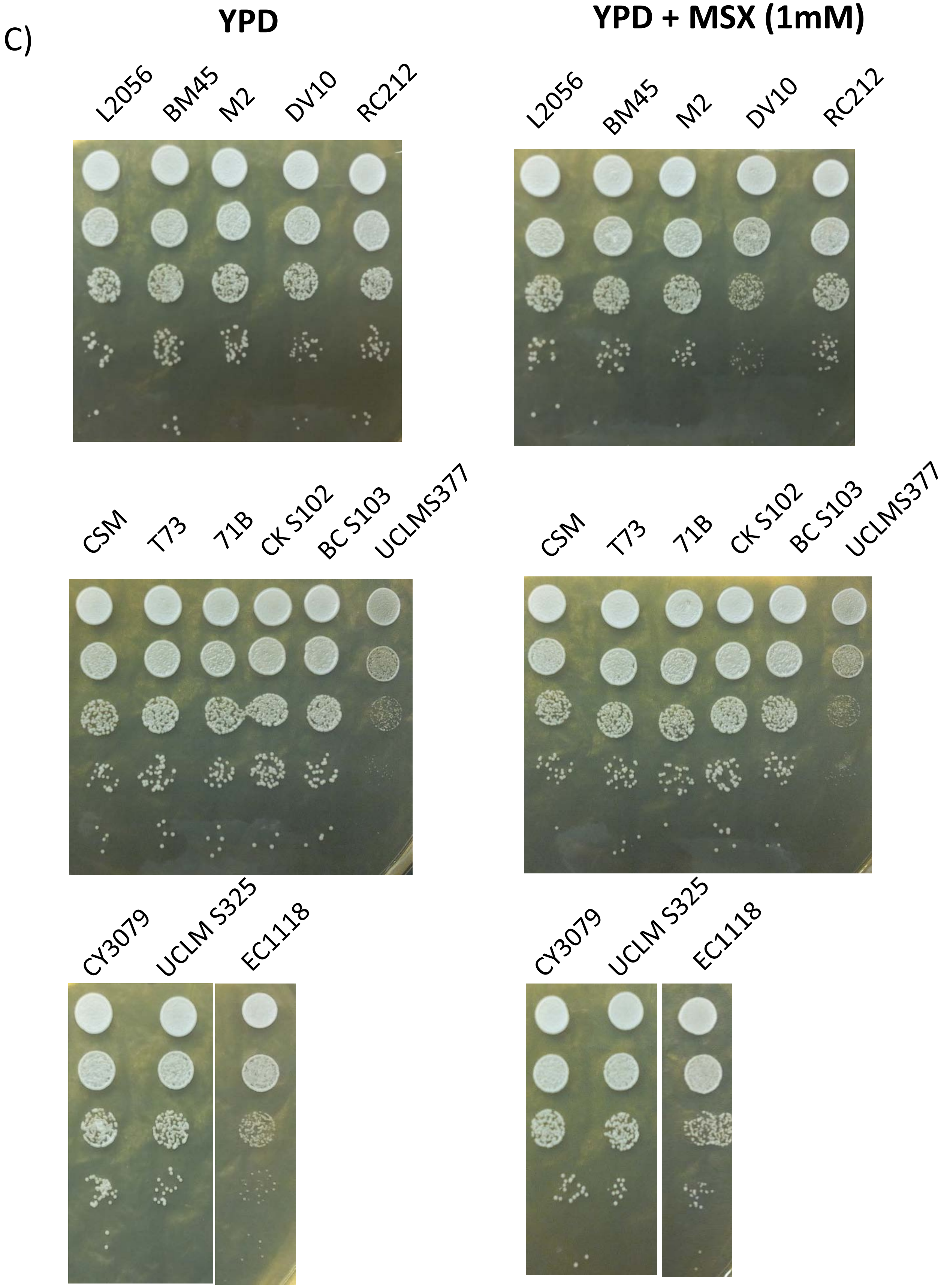
C)
YPD
YPD + MSX (1mM)
L2056
BM45
M2
DV10
RC212
CSM
T73
71B
CK S102
BC S103
UCLMS377
CY3079
UCLM S325
EC1118

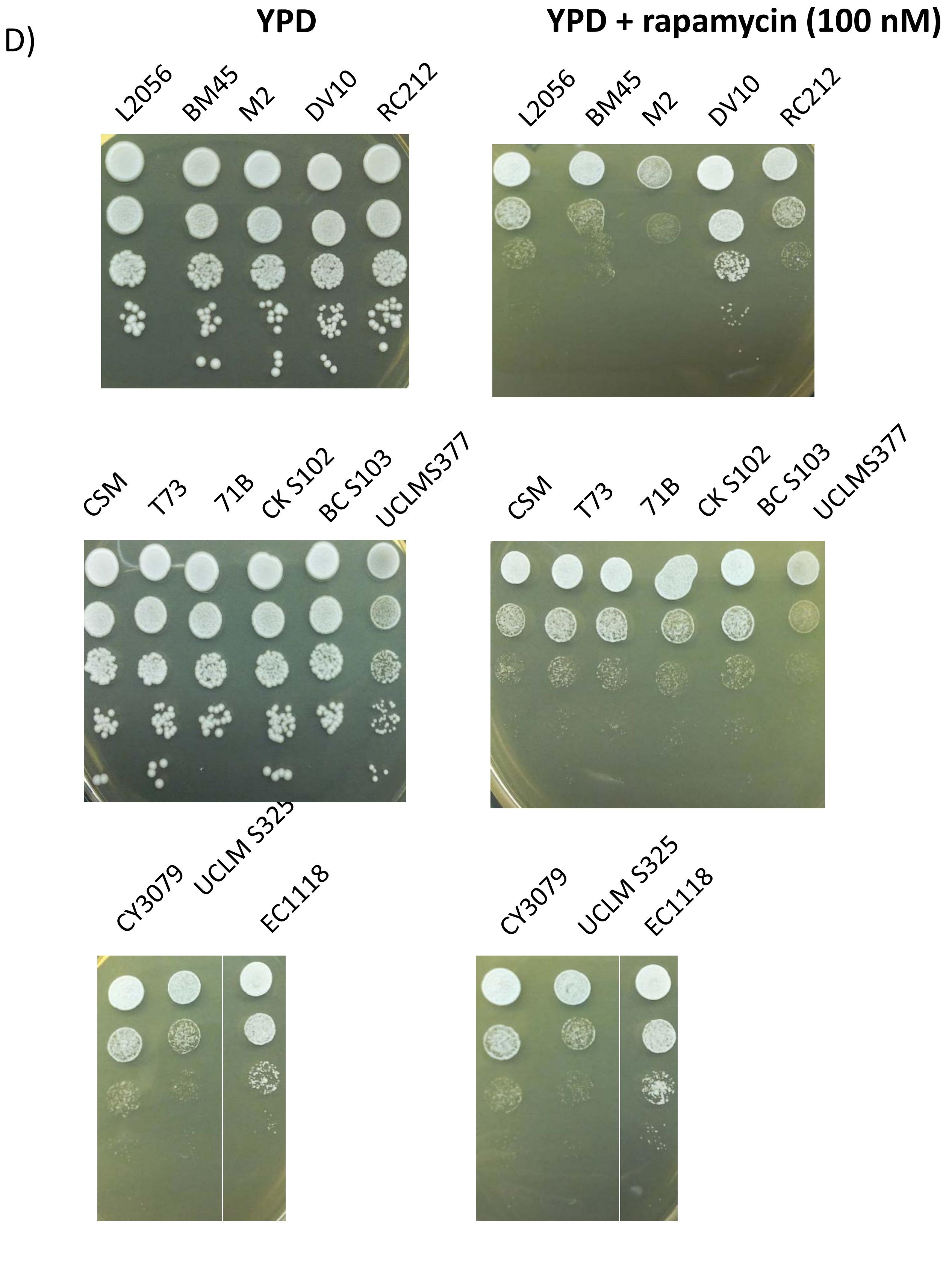

Additional File 2. Spot analysis of 14 commercial *S. cerevisiae strains*. Serial dilutions were spotted on plates containing A) 60 mg/l canavanine on SD B) 1 mg/l sulfometuron methyl in SD C) 1 mM methionine sulfoximine in YPD D) 100 nM rapamycin in YPD
