# Supplementary material for: Saccharomyces cerevisiae nutrient signaling pathways show an unexpected early activation pattern during winemaking
Source: Microb Cell Fact. 2020 Jun 6;19:124. doi: 10.1186/s12934-020-01381-6 (PMC7275465; doi:10.1186/s12934-020-01381-6)
Supplement: Supplementary file 3 — Additional file 3. Superoxide levels measured by dihydroethidium (DHE) incubation. Cells of selected strains were grown in YPD and tested in exponential and stationary condition. [file 12934_2020_1381_MOESM3_ESM.pdf]

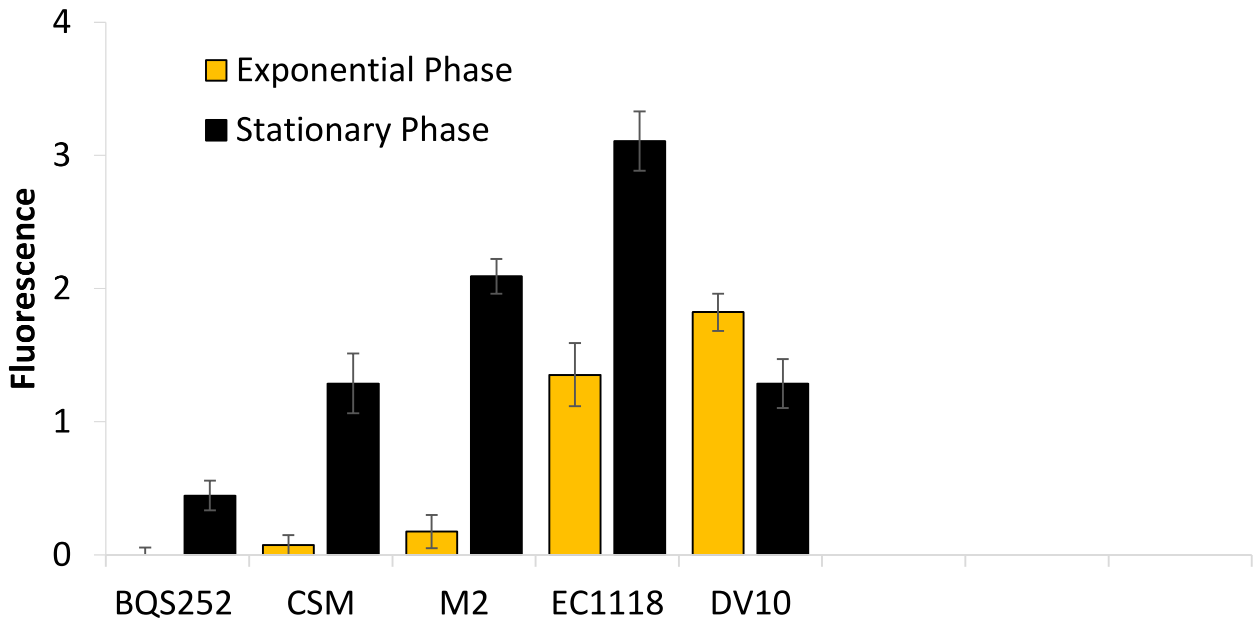

Additional File 3. Superoxide levels measured by dihydroethidium (DHE) incubation. Cells of selected strains were grown in YPD and tested in exponential and stationary condition.
