# Supplementary material for: Saccharomyces cerevisiae nutrient signaling pathways show an unexpected early activation pattern during winemaking
Source: Microb Cell Fact. 2020 Jun 6;19:124. doi: 10.1186/s12934-020-01381-6 (PMC7275465; doi:10.1186/s12934-020-01381-6)
Supplement: Supplementary file 4 — Additional file 4. Western blot analysis of PKA targets during MS300 fermentation for one day. Haploid C9 strains and mutants in potential PKA targets were used. Membrane was probed with an anti-PKA phosphorylation consensus RRXT/S. [file 12934_2020_1381_MOESM4_ESM.pdf]

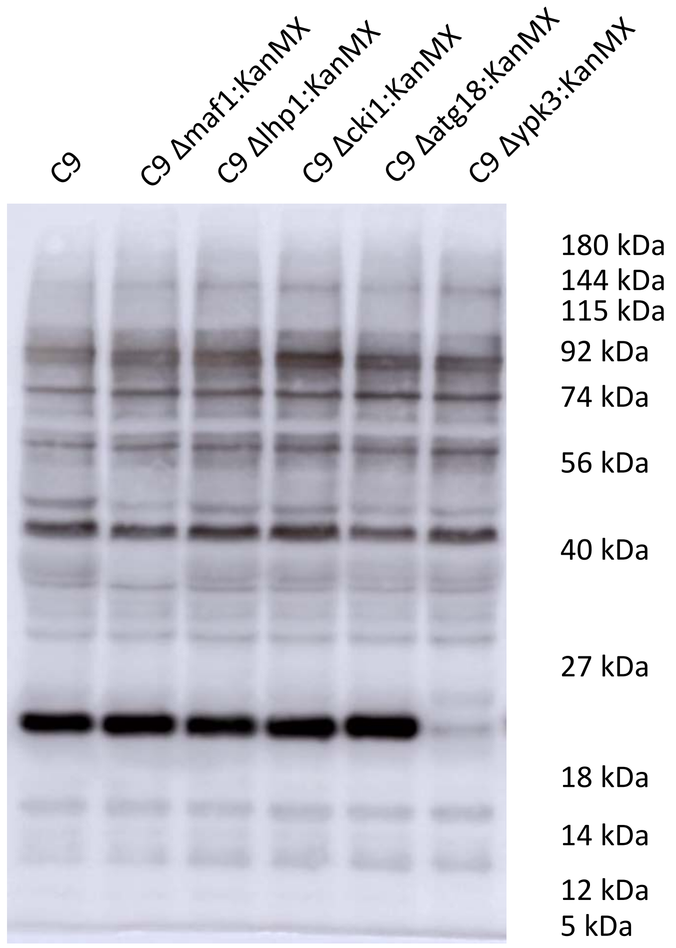

Additional File 4. Western blot analysis of PKA targets during MS300 fermentation for one day. Haploid C9 strains and mutants in potential PKA targets were used. Membrane was probed with an anti-PKA phosphorylation consensus RRXT/S.
