# Supplementary material for: Saccharomyces cerevisiae nutrient signaling pathways show an unexpected early activation pattern during winemaking
Source: Microb Cell Fact. 2020 Jun 6;19:124. doi: 10.1186/s12934-020-01381-6 (PMC7275465; doi:10.1186/s12934-020-01381-6)
Supplement: Supplementary file 5 — Additional file 5. Antibodies used in this work. [file 12934_2020_1381_MOESM5_ESM.pdf]

Additional File 5. Antibodies used in this work**.**

**Primary antibody**	**Source**	**Dilution**	**Secondary antibody**	**Source**	**Dilution**
Anti-Adh	Acris R1049	1:1000	Anti-rabbit	Santa Cruz Biotechnology	1:10000
Anti-MyC	Santa Cruz Biotechnology	1:1000	Anti-mouse	Santa Cruz Biotechnology/BioRad	1:10000/1:3000
Anti-Rps6 (S235-236)	Cell Signaling Technologies	1:1000	Anti-rabbit	Santa Cruz Biotechnology/BioRad	1:10000/1:3000
Anti-Rps6	Cell Signaling Technologies	1:1000	Anti-mouse	Santa Cruz Biotechnology/BioRad	1:10000/1:3000
Anti-Pgk1	Invitrogen	1:1500	Anti-rabbit	BioRad	1:3000
Anti-AMPKα (Thr172)	Cell Signaling Technologies	1:1000	Anti-rabbit	BioRad	1:3000
Anti-polyHis	Sigma-Aldrich (Merck)	1:3000	Anti-mouse	BioRad	1:5000
Anti-PKA consensus (RRXS*/T*)	Cell Signaling Technologies	1:1000	Anti-rabbit	BioRad	1:3000
